# Supplementary material for: Modes of administering sexual health and blood-borne virus surveys in migrant populations: A scoping review
Source: PLoS One. 2020 Aug 3;15(8):e0236821. doi: 10.1371/journal.pone.0236821 (PMC7398552; doi:10.1371/journal.pone.0236821)
Supplement: S1 Table — (DOCX) [file pone.0236821.s002.docx]

S1 Table. Search strategy for scoping review, by concept and database

| **Database** | **Concept 1 (migrants)** | **Concept 2 (surveys)** | **Concept 3 (administration mode)** |
| --- | --- | --- | --- |
| Embase | - exp communication barrier/ or exp language/ - exp migration/ - exp migrant/ - exp refugee/ - (((language$ or communicat$) and (barrier$ or understand$ or strateg$ or proficien$)) or translat$ or interpret$ or (cultur$ and competen$)).ti,ab. - (illiteracy or illiterate$).ti,ab. - (immigrant$ or migrant$ or asylum or refugee$ or undocumented).ti,ab. - (displaced and (people or person$1)).ti,ab. - (born adj2 overseas).ti,ab. - ("culturally and linguistically diverse" or CALD).ti,ab. - "foreign born".ti,ab. | - exp health care survey/ or exp health survey/ or exp questionnaire/ - exp self report/ - exp cross-sectional study/ - survey.ti,ab. - questionnaire.ti,ab | - ("face to face" or "face-to-face" or in-person or "in person").ti,ab. - web-based.ti,ab. - ((online adj5 survey) or (online adj5 questionnaire)).ti,ab. - (ipad or tablet or "personal digital assistant" or PDA or "handheld device" or "hand-held device" or "electronic device").ti,ab. - ("paper adj3 survey" or "pen adj2 paper" or handwritten or print$).ti,ab. - ((mode or method or way) adj3 (administ$ or conduct$ or carr$ or complet$)).ti,ab. - (teleph$ or phone$).ti,ab. |
| Medline | - exp Communication Barriers/ - exp Language/ - exp "Emigration and Immigration"/ - exp "Emigrants and Immigrants"/ - exp "Transients and Migrants"/ - exp Refugees/ - (((language$ or communicat$) and (barrier$ or understand$ or strateg$ or proficien$)) or translat$ or interpret$ or (cultur$ and competen$)).ti,ab. - (illiteracy or illiterate$).ti,ab. - (immigrant$ or migrant$ or asylum or refugee$ or undocumented).ti,ab. - (displaced and (people or person$1)).ti,ab. - (born adj2 overseas).ti,ab. - ("culturally and linguistically diverse" or CALD).ti,ab. - "foreign born".ti,ab. | - exp "Surveys and Questionnaires"/ - exp Health Surveys/ - exp Health Care Surveys/ - exp Self Report/ - exp Cross-Sectional Studies/ - survey.ti,ab. - questionnaire.ti,ab. |  |
| Web of Science (Core Collection) | - TS=(((language$ or communicat$) and (barrier$ or understand$ or strateg$ or proficien$)) or translat$ or interpret$ or (cultur$ and competen$)) - TS=(illiteracy or illiterate*) - TS=(immigrant* or migrant* or asylum or refugee* or undocumented) - TS=(displaced and (people or person*)) - TS=(born NEAR/2 overseas) - TS=("culturally and linguistically diverse" or CALD) - TS="foreign born" | - TS=(Cross-Sectional OR survey OR questionnaire) | - TS=("face to face" or "face-to-face" or in-person or "in person") - TS=web-based - TS=((online NEAR/5 survey) or (online NEAR/5 questionnaire)) - TS=(ipad or tablet or "personal digital assistant" or PDA or "handheld device" or "hand-held device" or "electronic device") - TS=("paper NEAR/3 survey" or "pen NEAR/2 paper" or handwritten or print*) - TS=((mode or method or way) NEAR/3 (administ* or conduct* or carr* or complet*)) - TS=(teleph* or phone*) |
