# Supplementary material for: Modes of administering sexual health and blood-borne virus surveys in migrant populations: A scoping review
Source: PLoS One. 2020 Aug 3;15(8):e0236821. doi: 10.1371/journal.pone.0236821 (PMC7398552; doi:10.1371/journal.pone.0236821)
Supplement: S2 Table — (DOCX) [file pone.0236821.s003.docx]

**S2 Table:** Architecture for Excel Data Charting Table

| **Category** | **Sub-category** | **Open field response** |
| --- | --- | --- |
| General information | Title of the study |  |
|  | Year of publication |  |
|  | Author of the study |  |
|  | Country in which the study [was] conducted |  |
| Study details | Survey topic |  |
|  | Sample frame |  |
|  | Survey setting |  |
|  | Survey technique/mode |  |
|  | Duration of the survey (in months) |  |
| Participant details | Male (nos) |  |
|  | Female (nos) |  |
|  | Transgender |  |
|  | Age group (years) |  |
|  | Length of stay in the country (years) |  |
| Survey methods | Who conducted the survey |  |
|  | Training (in case of interviewer who carried out the survey) |  |
|  | Language used in the survey |  |
|  | Language used in the interview |  |
|  | Interview setting |  |
|  | Duration of interview (in minutes) |  |
|  | No. of questions |  |
|  | Time taken to complete the survey |  |
|  | Pilot testing |  |
| Findings | Sample size |  |
|  | Response rate |  |
|  | Recruitment method |  |
|  | Missing data |  |
| Outcome | Factors that facilitated survey administration |  |
|  | Barriers |  |
|  | Recommendations |  |
| Note |  |  |
