# Supplementary material for: Modes of administering sexual health and blood-borne virus surveys in migrant populations: A scoping review
Source: PLoS One. 2020 Aug 3;15(8):e0236821. doi: 10.1371/journal.pone.0236821 (PMC7398552; doi:10.1371/journal.pone.0236821)
Supplement: S3 Table — (DOCX) [file pone.0236821.s004.docx]

**S3 Table:** Architecture for Excel Data Charting Table

| **Category** | **Data entry options** |
| --- | --- |
| Author, year | Open field |
| Study location (country) | Open field |
| Sample frame (region migrants were from) | Drop-down choices:   - Africa - Asia - Europe (Central/Eastern) - Latin America - Middle East - Other |
| Survey topic | Drop-down choices:   - Knowledge, behaviours and attitudes - Access to health services - Evaluation of intervention - Other |
| Survey technique / mode | Drop-down choices:   - Face-to-face - Paper and pen - Computer assisted self-interview - Hand-held electronic device - Online - Phone interview - Other |
| Sample size (of migrants) | Drop-down choices:   - 1-100 - 101-200 - 201-300 - 301-400 - 401-500 - 501-600 - 601-700 - 701-800 - 801-900 - 901-1000 - 1001-2000 - 2001+ |
| Response rate | Open field |
| Recruitment method | Drop-down choices:   - Community centres/groups - Email - Webpage - Database/med records - Intercept - Radio/tv - Snowball/word of mouth - Written invitation - Peer recruiter - Social media - Print media - Other |
| Incentives offered | Drop-down choices:   - Yes - Not stated |
| Survey pilot tested | Drop-down choices:   - Yes - No - Not reported |
| Survey available in respondent’s first language | Drop-down choices:   - Yes - No - Not reported |
| Length of survey | Drop-down choices:   - <10 min - 10-19 min - 20-29 min - 30-39 min - 40+ min - Not reported |
| If face-to-face interview, where conducted | Open field |
| Reported strengths/facilitators of survey administration methods | Open field |
| Reported weaknesses/barriers of survey administration methods | Open field |
| Any recommendations for future survey administration techniques | Open field |

**NOTE: If more than one drop-down choice applied, enter each individually in a separate row**
