# Supplementary material for: Modes of administering sexual health and blood-borne virus surveys in migrant populations: A scoping review
Source: PLoS One. 2020 Aug 3;15(8):e0236821. doi: 10.1371/journal.pone.0236821 (PMC7398552; doi:10.1371/journal.pone.0236821)
Supplement: Attachment — Submitted filename: PLOS One_review.docx [file pone.0236821.s005.docx]

This paper was interesting to me as a migration and health researcher. We have debated the ethics of asking migrants about sexual health, in particular the ethical challenges of taking blood samples for diagnoses, particularly in countries where BBV are deportable conditions (e.g. Malaysia, Singapore).

My main concern with this paper is in the reporting. The authors should be aware that there is a PRISMA extension for Scoping reviews.

PRISMA-ScR checklist: <http://www.prisma-statement.org/Extensions/ScopingReviews>

2018 Tricco elaboration article: <https://annals.org/aim/fullarticle/2700389/prisma-extension-scoping-reviews-prisma-scr-checklist-explanation>

Using an earlier methodological paper defining scoping reviews from Arksey and O’Malley is a limitation. As the PRISMA-ScR was not followed, I suggest that the authors state this as a Limitation in the Discussion section. Alternatively, the authors can consider re-writing the paper to follow PRISMA guidelines. The foundation and steps followed are in the manuscript, but it does require some rewriting and expansion of the Methods section, to include details like were data extraction forms piloted and by whom, etc.

My second comment that applies to the overall manuscript, is that the authors sufficiently caveat, in the Introduction and Discussion, that mode of survey administration is one of numerous factors affecting data quality. Specific suggestions are offered below.

**Methods:**

l. 107 – please insert a brief explanation describing what domains the Arksey and O’Malley framework covers. Here, authors should explicitly state that the PRISMA-ScR was not followed and the reason for this. The authors can consider including a figure depicting the Arksey and O’Malley domains for ease of reading.

l. 119 – after describing the objectives, the inclusion/exclusion criteria (and correspondingly Table 2) should be placed before information on searches and search strings. This is the preferred order in PRISMA reviews, with the reason that we need to see the eligibility criteria before assessing search terms etc against them. I’d suggest including some brief narrative text on inclusion/exclusion criteria alongside Table 2.

l. 136 – Bramer and Giustini framework – suggest to add ‘for this purpose’ at end of the sentence. Generally when introducing a framework, a brief explanation of its content will help readers who are not familiar with these articles.

**Discussion:**

l. 268 – Citing study specific reporting guidelines would be helpful after ref. 113, e.g. STROBE for observational studies. List here: <https://www.equator-network.org/>

l. 273 – on conducting surveys more efficiently and effectively to produce reliable data - Mode of administration is just one factor affecting response rates. Other factors include how survey constructs were developed and validated in local settings, whether it was piloted and cultural/linguistic adjustments made, inter-rater reliability (e.g. enumerator training to ensure standardized answers across enumerators) and sufficient ethical procedures to ensure participants that data collected is confidential, anoynmized, etc. For patient reported outcome measures (PROMS), there is something called the COSMIN standards which include a 114-item checklist to assess content validity of outcome measures, with a section on reliability. Line 288 goes on to ask whether methods produced valid and reliable data. To assess this comprehensively, we need to conduct critical appraisal at the level of the individual study (e.g using. JBI, CASP tools) and at the level of the outcome measure (e.g. COSMIN tool, or shorter appraisal checklists which examine outcome measures only, e.g. 5 item checklist in this [article](https://www.thelancet.com/journals/lanpsy/article/PIIS2215-0366(16)30047-5/fulltext#seccestitle30)). I’d suggest the authors refer to critical appraisal tools in this section, to offer specific guidance to researchers looking to improve the reliability and validity of survey data they collect with migrant populations.

Whether the included studies used validated SRH or SHBBV survey modules, vs. researchers making up their own questions, is not specified. This is especially important given the sensitive nature of the research topic. Using validated measures can help generate reliable data. The authors should mention somewhere in the Discussion, the rough proportion of studies which used validated (or at least, established) measures, and what the most commonly used measures are called/which larger surveys they were taken from (e.g. NATSAL in the UK/DHS globally. Other example measures on [p.4 in this document](https://drive.google.com/file/d/1f33Eustjgoqq4cY34kknc9UMvw_NIP3c/view)). Were any of the measures validated with migrant populations as well?

Searching online, I was shocked to see there is no internationally validated or recommended instrument (<https://www.who.int/docs/default-source/reproductive-health/sexual-health-survey-instrument-info-faq.pdf?sfvrsn=3172d357_2>) – I’d suggest that the authors mention that the WHO is actively soliciting submissions from researchers to recommend appropriate survey modules, in the Discussion.

l. 345 recommendation 4 – suggest to include that authors specify that culturally appropriate adjustments are made (where needed) after piloting surveys.

l. 355 – reference to better reporting – suggest to cite STROBE, CONSORT etc specifically to help guide researchers in the right direction.

l. 352 – the Limitation section should be expanded with the main limitation that PRISMA-ScR guidelines were not followed. While not required for scoping reviews, the lack of critical appraisal (at the level of the individual study, and outcome measure) can be considered a limitation, given that reliability of data will be affected by study design decisions, and content validity of constructs in survey modules.

**PRISMA flow diagram** – if available, authors should insert specific reasons for exclusion at the full-text stage.
